# Lipid Mediator Profiles Predict Response to Therapy with an Oral Frankincense Extract in Relapsing-Remitting Multiple Sclerosis

**DOI:** 10.1038/s41598-020-65215-6

**Published:** 2020-05-29

**Authors:** Klarissa Hanja Stürner, Oliver Werz, Andreas Koeberle, Markus Otto, Ole Pless, Frank Leypoldt, Friedemann Paul, Christoph Heesen

**Affiliations:** 1 Department of Neurology, Christian-Albrechts-University, Rosalind Franklin Straße 10, Kiel, Germany; 2 Department of Pharmaceutical/Medicinal Chemistry, Institute of Pharmacy, Friedrich Schiller University Jena, Philosophenweg 14, 07743 Jena, Germany; 3 Michael Popp Research Institute, University of Innsbruck, Mitterweg 24, 6020 Innsbruck, Austria; 4 Department of Neurology, Ulm University, Oberer Eselsberg 45, 89081 Ulm, Germany; 5 Fraunhofer IME, ScreeningPort, Schnackenburgallee 114, 22525 Hamburg, Germany; 6 Institute of Clinical Chemistry, Kiel University, 24105 Kiel, Germany; 7 Experimental and Clinical Research Center, Max Delbrueck Center for Molecular Medicine and Charité – Universitätsmedizin Berlin, Berlin, Germany; 8 NeuroCure Clinical Research Center, Charité – Universitätsmedizin Berlin, corporate member of Freie Universität Berlin, Humboldt-Universität zu Berlin, and Berlin Institute of Health, Berlin, Germany; 9 Institute of Neuroimmunology and Multiple Sclerosis, Center for Molecular Neurobiology Hamburg, University Medical Center Hamburg-Eppendorf, Hamburg, Germany

**Keywords:** Predictive markers, Drug development, Multiple sclerosis

## Abstract

Lipid mediators (LMs) are a unique class of immunoregulatory signalling molecules and known to be affected by frankincense extracts. We performed LM profiling by metabololipidomics in plasma samples from 28 relapsing-remitting multiple sclerosis (RR-MS) patients who took a standardised frankincense extract (SFE) daily for eight months in a clinical phase IIa trial (NCT01450124) and in 28 age- and gender-matched healthy controls. Magnetic resonance imaging, immunological outcomes and serum neurofilament light chain levels were correlated to changes in the LM profiles of the RR-MS cohort. Eight out of 44 analysed LMs were significantly reduced during an eight-month treatment period by the SFE and seven of these eight significant LM derive from the 5-lipoxygenase (5-LO) pathway. Baseline levels of 12- and 15-LO products were elevated in patients who exhibited disease activity (EDA) during SFE treatment compared to no-evidence-of-disease-activity (NEDA) patients and could predict treatment response to the SFE in a prediction model at baseline. Oral treatment with an SFE significantly reduces 5-LO-derived LMs in RR-MS patients during an eight-month treatment period. Treatment response to an SFE, however, seems to be related to 12-,15-LO and cyclooxygenase product levels before SFE exposure. Further studies should confirm their biomarker potential in RR-MS and SFE treatment.

## Introduction

Multiple sclerosis is the most common debilitating chronic autoimmune disease of the central nervous system affecting more than 2 million predominantly young female adults worldwide^1^. Oral drugs exhibiting a favourable safety profile for the treatment of relapsing-remitting multiple sclerosis (RR-MS) are of high interest for patients and treaters because of the treatment’s long-term perspective in RR-MS. Boswellic acids (BAs), the main biologically active ingredients of frankincense, are orally available and exhibit anti-inflammatory activities in combination with beneficial data on safety and tolerability^2,3^. Of these BA-associated anti-inflammatory responses, the best described are pharmacologically induced changes of the lipid mediator (LM) profile, in particular the inhibition of 5-lipoxygenase (5-LO)^2^, an enzyme which has been implicated in MS pathogenesis due to its expression in RR-MS brain lesions^4^. Little is known about the impact of LMs and their relevance to MS pathology, however accumulating data indicates that the importance of LMs has been underestimated in MS to date^5^ and that LM-producing enzymes are involved in disease pathogenesis and progression^6,7^. Consequently, we analysed the LM profile in RR-MS patients before and after oral BA-treatment with an SFE for eight months in a clinical phase IIa trial.

## Material and Methods

We performed a bicentric, phase IIa, open-label, baseline-to-treatment trial between 2011 and 2017 with an orally available SFE produced by Alpinia Institute for Life Sciences AG (Walenstadt, Switzerland) at two German tertiary academic MS centers (SABA Trial^8^, NCT01450124). The study was approved by the German Federal Institute for Drugs and Medical Devices (BfArM, Approval No. 4036771), and by the local ethics committees (Ethik-Kommission der Ärztekammer Hamburg, Approval No. PVN3389 and PVN2758 and Ethikkommission der Charité – Universitätsmedizin Berlin, Approval No. ZS EK 13572/09). All participants gave their written informed consent at screening and the study and all experiments were conducted in accordance with the Declaration of Helsinki and with good clinical practice. Plasma and serum samples were collected before the start of treatment and at month 8, respectively, and stored at −80 °C, as described previously^8^. Samples originated from n = 28 RR-MS patients who took 3600 to 4800 mg of an SFE daily and from 28 age- and gender-matched healthy controls. LM profiling by metabololipidomics was performed by analysing a panel of 44 LMs using ultraperformance liquid chromatography ESI tandem mass spectrometry (UPLC-MS-MS)^9^. Signal intensities (peak areas) were normalized to the internal standard PGB1 to correct for differences in sample preparation.

Statistics were carried out using R^10^ and the packages ggplot2^11^ and corrgram^12^. For calculating the fold-increase in RR-MS patients versu healthy controls the median value for each LM of the healthy control cohort was used as normalization value. LM analysis of longitudinal samples was performed using paired Wilcoxon tests (baseline vs. follow-up) with Bonferroni adjustment. LMs were also correlated with magnetic resonance imaging (MRI), immunological, biomarker outcomes (collected as described previously^8^) and serum neurofilament light chain (NfL) concentrations. NfL and Glial fibrillary acidic protein (GFAP) were measured using the Simoa NF-light® assay (Quanterix, USA). For analysing correlations, descriptive statistics with a Spearman’s of p < 0.05 were used. For further analysis, patients in the SABA trial were subdivided according to no-evidence-of-disease activity (NEDA) criteria (defined by lack of a relapse, lack of new T2 lesions and new contrast-enhancing lesions (CEL) and no confirmed disease progression (NEDA3)) into a NEDA group and an evidence of disease activity (EDA) patient group. EDA patients had at least either a relapse, a new T2 lesion or CEL or a confirmed disease progression in EDSS during the treatment phase of the trial. Combined analysis for prediction of NEDA status using LM expression was performed using the Combiroc Online Tool^13^.

## Results

### Lipid mediator expression differs between healthy controls and MS patients

**Figure 1:**
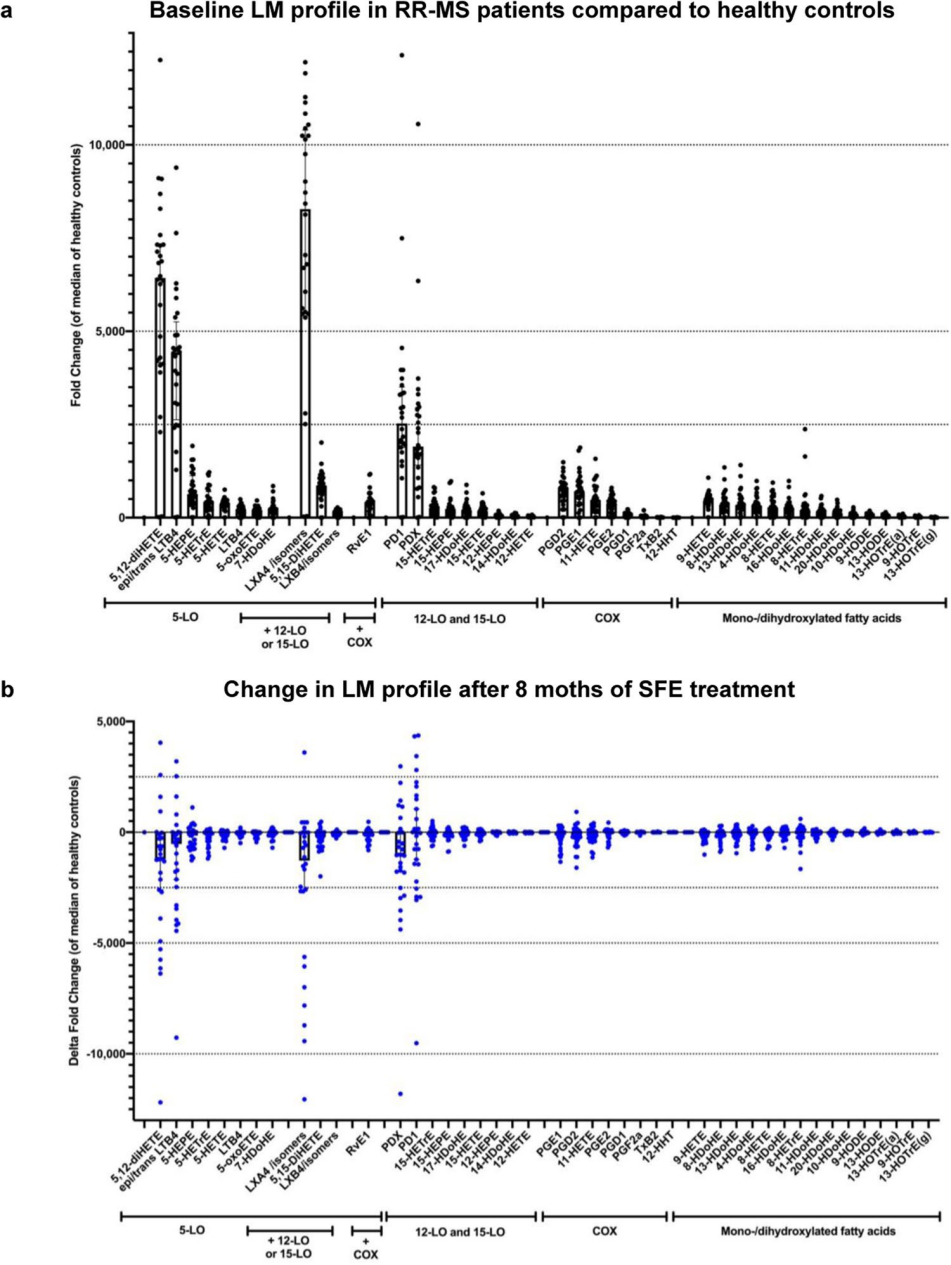
Lipid mediator profile in 28 untreated RR-MS patients (black, upper panel a) and changes (delta values) in the identical patient cohort after 8 months of treatment with a standardised frankincense extract (blue, lower panel b). Individual data are plotted as dots with median and interquartile range, in the lower panel data are presented as median delta values from baseline with interquartile range.

Except for 12-HHT, measured LMs were significantly higher in RR-MS patients compared to healthy age- and gender-matched controls with the topmost fold-increase (>1000-fold) in five LMs being: the three 5-LO-derived LM lipoxin A_4_ and/or isomers, 5,12-di-hydroxyeicosatetraenoic acid (5,12-diHETE), leukotriene B_4_ (LTB_4_), and the two 12- or 15-LO-derived LM protectin D1 (PD1) and protectin DX (PDX). Thirty-one LMs (i.e. 70%) were >100-fold more abundant in RR-MS than in healthy donors (Fig. 1a, Supplemental Table S1).

### An eight-month treatment with an SFE changes the LM profile in RR-MS patients

**Figure 2:**
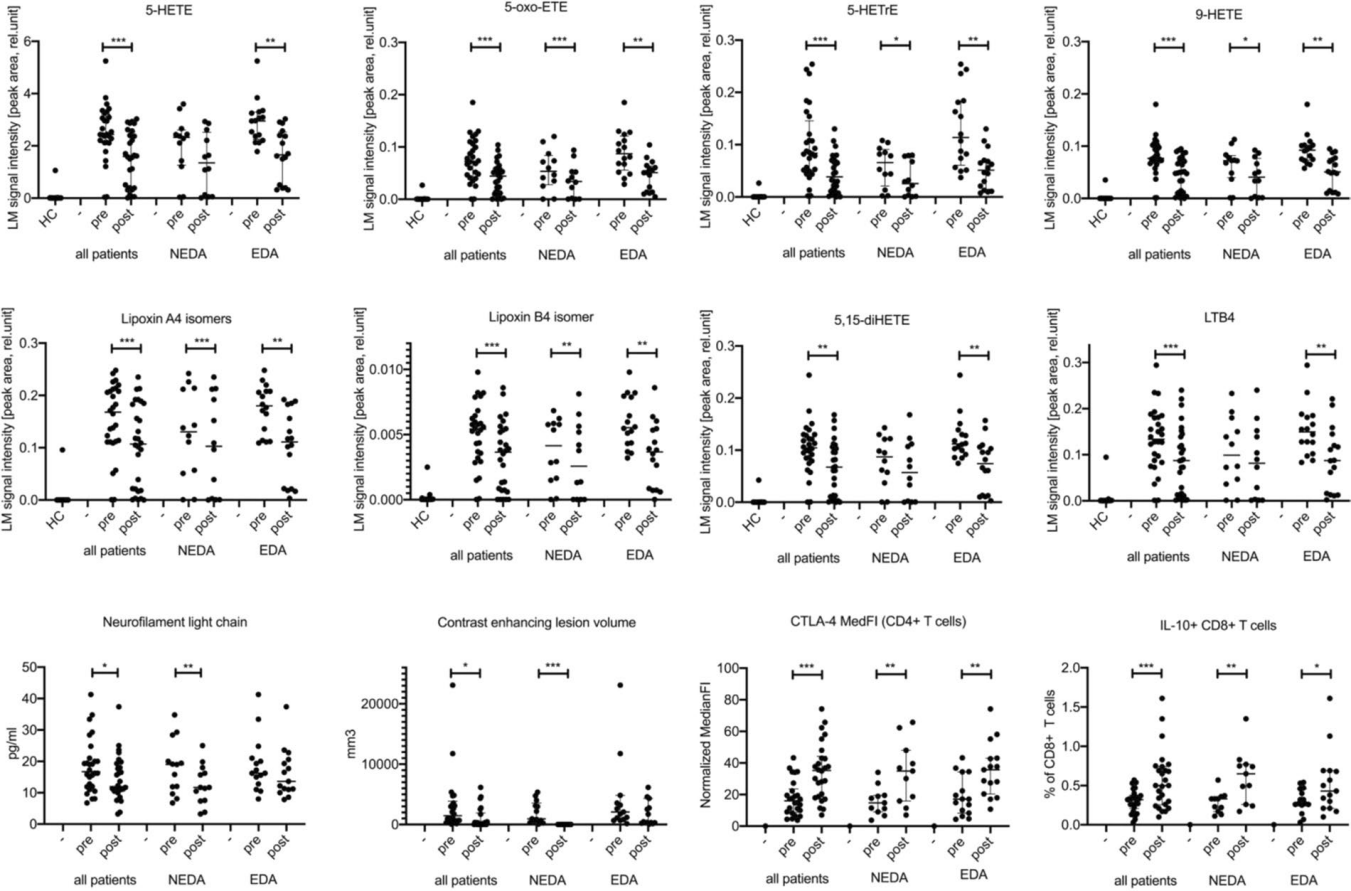
Treatment effect of a standardised frankincense extract (SFE) on selected lipid mediators, neurofilament light chain, contrast enhancing lesion volume, and immunological outcomes in the SABA trial (n = 28 patients; n = 12 NEDA and n = 16 EDA patients). HC: healthy controls. Pre: baseline values before start of treatment, post: values after 8 months of continuous SFE treatment. ***p < 0.001, **p < 0.01, *p < 0.05.

**Figure 3:**
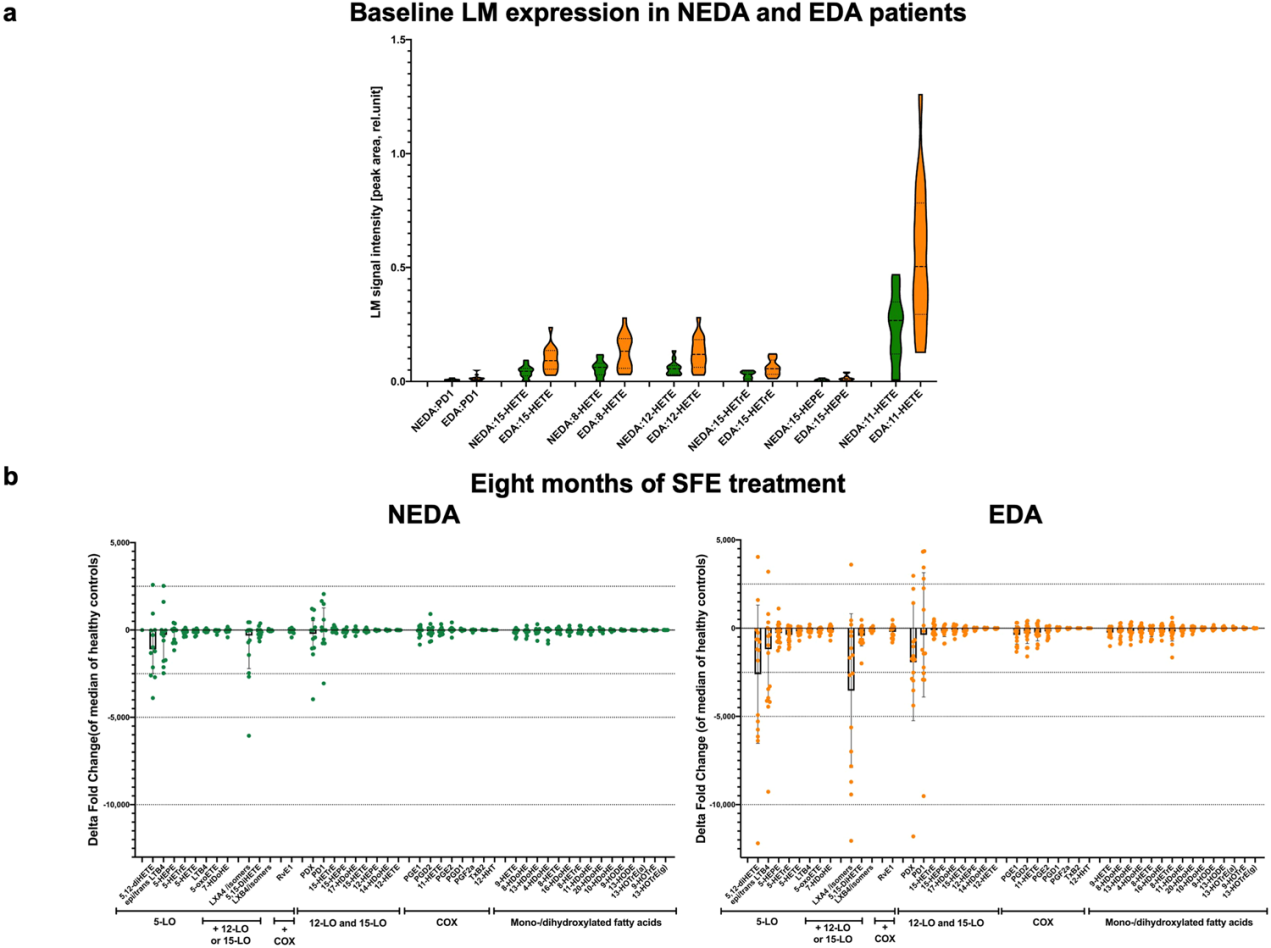
Upper Panel a: Baseline expression of selected LMs (p ≤ 0.01) in NEDA (in green, n = 12) versus EDA MS patients (orange, n = 16) of the SABA trial. LM expression is plotted as violin plots indicating median (dashed line) and interquartile range (dotted line) of the LM signal intensity measured as peak area. Lower Panel b: Changes (delta values) in NEDA (n = 12, green) versus EDA (n = 16, orange) SABA patients after eight months of treatment with a standardised frankincense extract. Individual data are plotted as median of delta values from individual baseline values of the fold increase versus healthy controls with interquartile range.

After receiving a SFE for eight months, a general decrease of LMs was observed (Figs. 1b and 2). However, only eight of 44 analysed LMs were significantly reduced in the 28 RR-MS patients. These were: 9-HETE, lipoxin A_4_ and/or isomers, lipoxin B_4_ and/or isomers, 5,15-diHETE, LTB_4_, 5-HETE and its metabolites 5-oxo-eicosatetraenoic acid (5-oxo-ETE), and 5*S*-hydroxy-6*E*,8*Z*,11*Z*-eicosatrienoic acid, (5-HETrE). Seven of these eight LMs are generated by the 5-LO pathway, while 9-HETE is predominantly produced by non-enzymatic oxidation of arachidonic acid. For seven of these LMs the reduction was more pronounced in EDA patients when compared to MS patients fulfilling NEDA criteria during SFE treatment (Figs. 2, 3b and Supplemental Table S2) thereby triggering further analyses of the NEDA and EDA subgroups. Only for 5-oxo-ETE, a bioactive metabolite of 5-HETE, the reduction during SFE treatment was more pronounced in NEDA patients (Fig. 2 and Supplemental Table 2).

### Baseline expression of 12- and 15-LO derived lipid mediators predicts treatment response to an SFE

**Figure 4:**
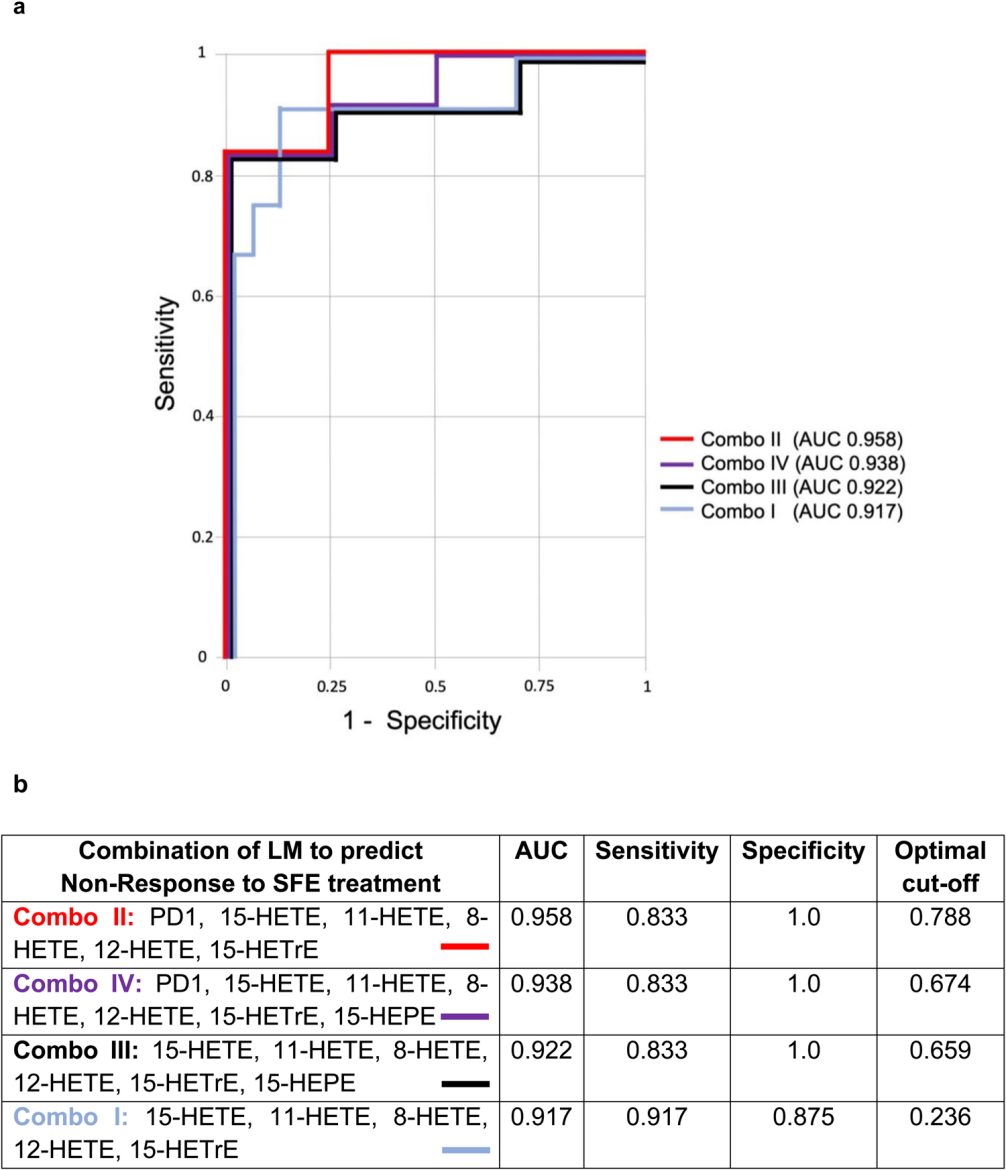
(**a**) Overlay of multiple ROC curves for the LMs of Fig. 3a in the CombiROC analysis. (**b**) Results from the CombiROC analysis for the prediction of EDA status/Non-Response to SFE treatment from baseline LM levels.

When we analysed the LM profile of NEDA and EDA patients of the trial cohort at baseline, certain LMs were already found to be increased in the trial subjects, later defined as EDA RR-MS patients (Fig. 3a). When analysing the 7 LMs with the lowest p-values (all p ≤ 0.01: PD1, 11-HETE, 12-HETE, 15-HETE, 8-HETE, 15-HETrE, 15-HEPE) for their performance in predicting the response to SFE treatment, the ROC curve yielded results of 83% for sensitivity and 100% for specificity (AUC 0.958, Fig. 4), indicating that these LMs could be useful as biomarkers to predict therapy response to an SFE prior to treatment. For the majority of these markers, 12- and 15-LOs are involved in their biosynthesis.

### LM correlations with biomarkers of MS are dependent on treatment response to an SFE

**Figure 5:**
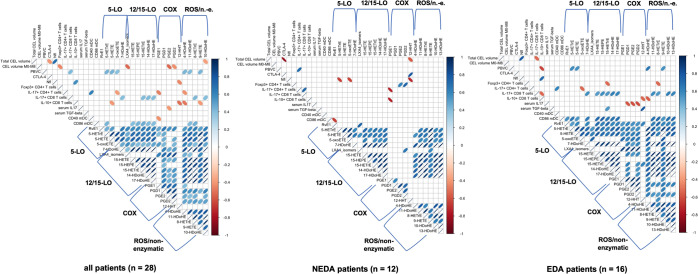
Correlation matrices of lipid mediators and biomarkers of the SABA trial; positive correlations are blue, negative correlations are red. Only correlations with a Spearman’s of p < 0.05 are plotted. The thinner the dot, the smaller the p-value. LMs are ordered by key enzyme. Left: total SABA patient cohort (n = 28), middle: NEDA patients (n = 12), right: active EDA RR-MS patients, i.e. Non-responders (n = 16). Exact values of the correlations are given in the Supplementary Material. CEL: contrast enhancing lesion, M0-M8: treatment phase of the trial.

Correlation analysis (Fig. 5, Suppl. Tables S3–5) between LM and MRI, immunological and NfL outcomes in the complete SFE-treated patient cohort revealed that LMs showed only moderate correlations in the total patient cohort. However, when the SFE-treated RR-MS patient cohort was split according to NEDA criteria, the correlation matrices revealed differing patterns in the NEDA versus EDA groups (Fig. 5, middle and right panel).

**Figure 6:**
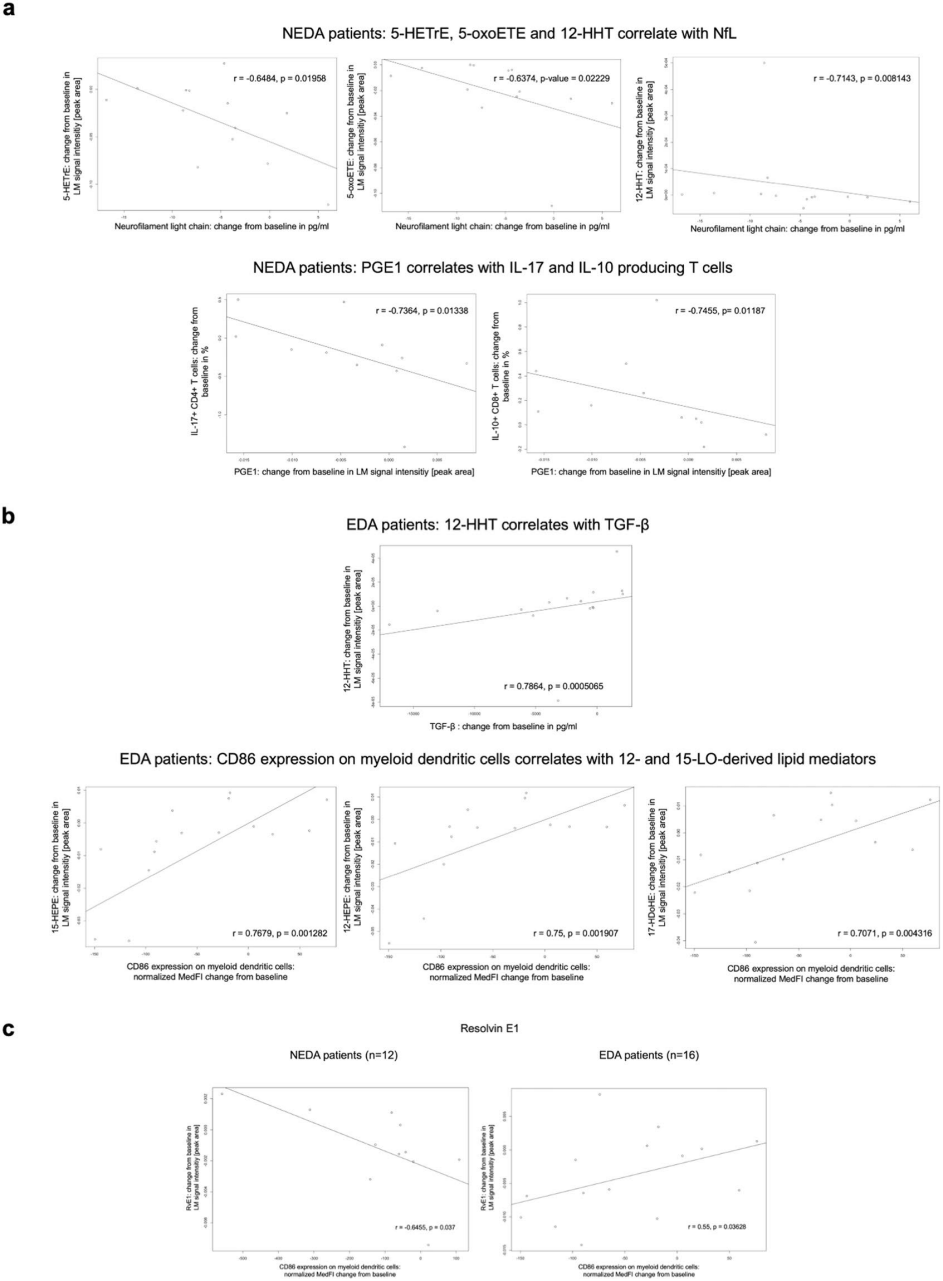
Strongest LM correlations in (**a**) NEDA and (**b**) EDA RR-MS patients of the SABA trial; (**c**) shows the correlation plots for Resolvin E1 in NEDA (left) versus EDA (right) RR-MS patients of the SABA trial.

For NEDA patients, three strong negative correlations involving LMs were apparent (Fig. 6a and Suppl. Table 4). These were: 1) PGE_1_ and the proportion of IL-10 positive CD8+ T cells (r = −0.754), 2) PGE_1_ and the proportion of IL-17+ CD4+ T cells (r = −0.736) and 3) CD86 expression on myeloid dendritic cells and the 5-LO-derived LM resolvin E1 (RvE1, r = −0.645). The significant strong positive correlations involving LMs in NEDA patients were changes in: 1) CTLA-4 expression and 12-HHT (r = 0.728) and 2) PGD_2_ and the proportion of Foxp3+ CD4+ T cells (r = 0.727). Intra-individual NfL concentration changes during the trial showed a strong negative correlation with the significantly decreased 5-LO products 5-HETrE (r = −0.637) and 5-oxo-ETE (r = −0.648) and additionally with 12-HHT (r = −0.714). These negative correlations between NfL and LMs were only seen in the NEDA patient group.

Contrastingly, positive correlations were found in EDA patients for TGF-β and 12-HHT and for several 12/15-LO products and CD86 surface expression on myeloid dendritic cells (Fig. 6b and Suppl. Table 5). Strong negative correlations were evident in EDA patients for serum IL-17 levels and CTLA-4 (r = −0.696), PGD_2_ (r = −0.615) and PGE_2_ (r = −0.604), respectively.

When comparing the correlation matrices of NEDA and EDA patients, the most prominent difference indicated that COX-derived LMs correlated with other LMs only in EDA patients and failed to do so in the NEDA patient cohort (Fig. 5). The most striking single difference between the NEDA and EDA subgroups during SFE treatment was a correlation between CD86 expression on myeloid dendritic cells and the 5-LO-derived LM RvE1 (Fig. 6C), which was moderate and positive in EDA RR-MS patients (r = 0.55) and strong and negative in NEDA patients (r = −0.645).

Irrespective of the analysed LMs prominent correlations among the other analysed biomarkers in the total SABA patient cohort were: 1) changes in NfL serum concentration and the CEL volume, 2) the proportion of IL-17+ CD4+ T cells and CD40 surface expression of myeloid dendritic cells and 3) changes in NfL serum concentration and the proportion of IL-10+ CD8+ T cells (Suppl. Figures 1–3). In NEDA patients changes in CTLA-4 expression on CD4+ T cells and the volume of CEL in MRI showed a strong correlation (r = −0.836, Suppl. Figure 4). GFAP serum levels remained unaltered during treatment (Suppl. Figure 5). Most of these observations confirm previous reports^8,14,15^ and can serve as a quality control of the data set.

## Discussion

This is the first longitudinal study of LMs in an RR-MS patient cohort, and the first longitudinal analysis of a correlation of LMs and several biomarkers including MRI, immune cell subsets and NfL in peripheral blood of RR-MS patients and in patients treated with an SFE. The significant upregulation of LMs in this RR-MS patient cohort compared to healthy controls suggests that LM pathways are involved in the immunopathology of MS. When analysing data from a small clinical phase IIa trial with a 5-LO-inhibiting SFE, we observed a significant decline in 5-LO products after an eight-month treatment period, although *in vitro* data previously implied that BAs have an IC_50_ for 5-LO that is unlikely to be reached *in vivo*^16^. The use of an extract containing different BAs and related bioactive triterpene acids might provide an explanation for this result by exhibiting synergistic effects and thereby more efficiently inhibiting 5-LO. However, despite this clear effect on 5-LO products, the treatment response to the SFE in RR-MS patients seems more complex: LMs form an elaborated network and inhibition of single enzymes skews the LM metabolome in a cell-specific fashion^17^, e.g. 5-LO inhibitors reduce leukotrienes in M1 but less so in M2 macrophages. Furthermore, MS itself is a heterogeneous disease or may even represent a group of diseases that exhibit differences in treatment response to several established MS disease-modifying therapies, complicating the analysis of patient responses and disease outcomes. Our observations suggest that the treatment response to an SFE is not associated with 5-LO inhibition in RR-MS. However, upregulation of 12- or 15-LO and COX activity in RR-MS might define an MS subtype that has a suboptimal response to SFE treatment. This matches previous observations of an involvement of the 12- or 15-LO and COX in more active RR-MS patients^18,19^. The strong positive correlation between 12/15-LO-derived LMs and the surface expression of CD86 in dendritic cells in the EDA RR-MS group is in line with previous animal studies showing that 12/15-LO regulates the maturation process of dendritic cells and its inhibition favours TH17 differentiation^20^. Such a skew towards TH17 differentiation in the active EDA RR-MS group is evident from the positive correlations of 12- or 15-LO-derived LM and IL-17+ CD8+ T cells (Fig. 5). Considering the positive correlations between serum IL-17 levels and prostaglandins that promote IL-17 production and TH17 expansion^21^, all these observations suggest the presence of an IL-17-driven phenotype in the active RR-MS cohort that is unaffected by oral SFE treatment.

5-LO and particularly the 12/15-LO-derived LM can act as agonists of the peroxisome proliferator-activated receptor-γ (PPARγ)^22,23^, a transcription factor with relevance to the pathogenesis of MS and exhibiting potential as a therapeutic target. Hence, 5-LO- or 12/15-LO-deficient mice show a significant exacerbation of the disease course during EAE^24^. Upregulation of the associated LMs might therefore represent an endogenous anti-inflammatory and neuroprotective mechanism in MS patients. As this effect is more pronounced in RR-MS patients with disease activity during SFE treatment, the increase of 5-LO and 12/15-LO-derived LM might indicate that PPARγ-signalling is already upregulated in non-responding patients and thereby indirectly revealing one potential mechanism of action of the SFE in this trial. The opposite correlations of the pro-resolving LM RvE1 and CD86 expression of myeloid dendritic cells in the NEDA and non-responding EDA RR-MS subgroup further support the idea of pre-existing MS subgroups or phenotypes in which different pathways are involved in disease pathogenesis. Thus, RvE1 has been previously described as being able to decrease disease severity by suppressing autoreactive T cells in an MS mouse model^25^.

The correlated changes in Foxp3 and CTLA-4 expression in and of CD4+ T cells, CD86 expression of myeloid dendritic cells and several COX-controlled LM in NEDA patients and their absence in EDA MS patients with higher COX-controlled LM levels could also indicate the involvement of another dioxygenase in active RR-MS patients. One such dioxygenase that is known to be inhibited by COX is indoleamine 2,3-dioxygenase (IDO), a key enzyme in the kynurenine pathway, which exhibits immune checkpoint function in MS^26,27^. A pre-existing enhancement of COX activation in active RR-MS patients is thus likely to lower IDO expression and thereby prevent an immune therapeutic effect of BAs in these patients, while in the NEDA patients, the lower COX activity allows BAs to take effect.

## Conclusion

Oral treatment with an SFE significantly reduces bioactive 5-LO-derived LMs, MRI disease activity, and peripheral neurofilament light chain concentrations in RR-MS patients during an eight-month treatment period. However, inhibition of 5-LO *per se* is not associated with a treatment response to an SFE. In EDA patients during SFE treatment, LMs produced by 12- or 15-LOs are primarily upregulated before the start of the treatment and might be useful as predictors of an SFE-mediated treatment response in the future. When comparing correlation plots of the parameters analysed in the total SABA patient cohort, in the NEDA responder subgroup and the EDA treatment failure patient subgroup, different correlation profiles can be detected indicating a difference in the regulation of 12/15-LO- and COX-derived LM in these respective subpopulations and their associated immunological profiles. Further studies should investigate the potential of LMs as biomarkers in RR-MS and the mechanism of frankincense extracts in the suppression of disease activity in RR-MS.

## Supplementary information

Supplemental Information.

## Data Availability

The datasets generated during and/or analysed during the current study are available from the corresponding author on reasonable request.
