# Supplementary material for: Lipid Mediator Profiles Predict Response to Therapy with an Oral Frankincense Extract in Relapsing-Remitting Multiple Sclerosis
Source: Sci Rep. 2020 May 29;10:8776. doi: 10.1038/s41598-020-65215-6 (PMC7260364; doi:10.1038/s41598-020-65215-6)
Supplement: Supplementary file 1 — Supplemental Information. [file 41598_2020_65215_MOESM1_ESM.pdf]

SUPPLEMENTAL INFORMATION

for the manuscript:

"Lipid mediator profiles predict response to therapy with an oral frankincense extract in relapsing-remitting multiple sclerosis"

by

Klarissa Hanja Stürner *, Oliver Werz, Andreas Koeberle, Markus Otto, Ole Pless, Frank Leypoldt, Friedemann Paul and Christoph Heesen

I. Abbreviations:

5-LO: 5-Lipoxygenase

5-HETE: 5-Hydroxyeicosatetraenoic acid

5-HETrE: 5*S*-Hydroxy-6*E*,8*Z*,11*Z*-eicosatrienoic acid

5-oxo-ETE: 5-Oxo-eicosatetraenoic acid

9-HETE: *9*-Hydroxy-5,7,11-eicosatetraenoate

COX: Cyclooxygenase

HETE: Hydroxyeicosatetraenoic acid

HETrE: Hydroxy-6*E*,8*Z*,11*Z*-eicosatrienoic acid

HoDHE: Hydroxydocosahexaenoic acid

HHT: Hydroxyheptadecatrienoic acid

IL: Interleukin

mDC: myeloid dendritic cell

pDC: plasmacytoid dendritic cell

MRI: Magnetic resonance tomography

NEDA: no evidence of disease activity
defined as no relapse, no new CEL, no new T2 lesion and no disease progression during follow-up (NEDA 3)

EDA: evidence of disease activity

defined as at least one relapse, one new CEL or T2 lesion or confirmed disease progression during follow-up

LXA_4: Lipoxin A_4

LXB_4: Lipoxin B_4

PD1: Protectin D1

PGE1 or 2: Prostaglandin E 1 or 2

PGD2: Prostaglandin D2

RvE1: Resolvin E1

ROC: Receiver-Operating-Characteristic

ROS: reactive oxygen species

RR-MS: Relapsing-remitting multiple sclerosis

SABA: Safety, tolerability and efficacy of a standardized frankincense extract in multiple sclerosis (Trial name, NCT01450124)

SFE: standardized frankincense extract

TGF-β: Transforming growth factor β

Supplemental Table S1: median and p-values of LM analysis of healthy controls and RR-MS patients at baseline and after SFE treatment. Median data are presented as LM signal intensitiy [peak area] or Fold Change (increase), respectively. Following Bonferroni adjustment all p < 0.001136 are significant and highlighted in bold print.

Lipid Mediator	Median of peak area (LM intensity) of healthy controls at baseline	Median of peak area (LM intensity) of RR-MS at baseline	Median of Fold Increase in RR-MS at baseline	HC versus RR-MS patients at baseline p-value	Median of peak area (LM intensity) of RR-MS after eight months of SFE treatment	Median of Fold Increase in RR-MS after eight months of SFE treatment	SFE treatment: Baseline versus month 8 in 28 RR-MS patients p-value	Key enzyme
5,12-diHETE	0.000426	2.74	6431.92	**4.202e-10**	1.845	4330.99	0.001741	5-LO
Epi/trans LTB4	0.000131	0.58	4427.48	**4.438e-10**	0.3585	2736.64	0.003739	5-LO
5-HEPE	0.000491	0.3105	632.38	**2.582e-10**	0.209	425.66	0.006965	5-LO
**5-HETrE**	0.000208	0.0836	401.68	**2.877e-10**	0.0385	185.10	**0.000306**	5-LO
**5-HETE**	0.00699	2.435	348.35	**2.582e-10**	1.595	228.18	**0.0008496**	5-LO
**LTB4**	0.000597	0.1335	223.62	**7.176e-10**	0.08735	146.31	**0.0003418**	5-LO
**5-oxo-ETE**	0.0004	0.0725	181.25	**1.582e-09**	0.0443	110.75	**5.53e-05**	5-LO
7-HDoHE	0.0000678	0.0148	217.55	**2.582e-10**	0.01035	152.65	0.04261	5-LO
**LXA4 isomers**	0.0000203	0.168	8275.86	**3.207e-10**	0.107	5270.94	**0.0006738**	5-LO + 12- or 15-LO
**5,15-diHETE**	0.000121	0.1045	863.64	**3.576e-10**	0.06735	556.61	**0.001065**	5-LO + 12- or 15-LO
**LXB4 isomers**	0.0000356	0.0054	152.25	**5.995e-09**	0.00365	102.53	**0.0009657**	5-LO + 12- or 15-LO
RvE1	0.0000176	0.0071	404.55	**7.573e-10**	0.00569	323.30	0.01362	5-LO + COX
PD1	0.00000391	0.0099	2529.41	**2.58e-10**	0.007695	1968.03	n.s.	12- or 15-LO
PDX	0.00000592	0.0113	1908.78	**4.941e-10**	0.008285	1399.49	n.s.	12- or 15-LO
15-HETrE	0.000148	0.0389	263.18	**3.981e-10**	0.02615	176.69	n.s.	12- or 15-LO
15-HEPE	0.0000413	0.0096	232.69	**2.879e-10**	0.005015	121.43	0.02041	12- or 15-LO
17-HDoHE	0.0000659	0.0136	205.61	**3.209e-10**	0.01056	160.24	n.s.	12- or 15-LO
15-HETE	0.000363	0.0639	176.03	**2.582e-10**	0.0406	111.85	0.04512	12- or 15-LO
12-HEPE	0.000338	0.0133	39.20	**6.12e-10**	0.00702	20.77	0.0221	12- or 15-LO

14-HDoHE	0.000464	0.0155	33.41	**9.36e-10**	0.0121	26.08	n.s.	12- or 15-LO
12-HETE	0.0036	0.0781	21.69	**7.269e-14**	0.04945	13.74	0.02475	12- or 15-LO
PGD2	0.0000228	0.0174	763.16	**9.936e-15**	0.0125	548.25	n.s.	COX
PGE1	0.0000229	0.0163	709.61	**1.951e-13**	0.010185	444.76	0.008225	COX
11-HETE	0.000798	0.3795	475.56	**3.772e-10**	0.202	253.13	0.04387	COX
PGE2	0.0000792	0.0298	376.26	**3.558e-10**	0.0215	271.46	n.s.	COX
PGD1	0.0000677	0.005	74.37	**2.656e-09**	0.004865	71.86	n.s.	COX
PGF2a	0.000109	0.003	27.89	**1.037e-09**	0.00278	25.50	n.s.	COX
TxB2	0.000988	0.0086	8.70	**1.348e-10**	0.007035	7.12	n.s.	COX
12-HHT	0.000028	0.000032	1.14	0.106	0.00002515	0.90	n.s.	COX
**9-HETE**	0.000168	0.0767	456.55	**2.582e-10**	0.04935	293.75	**0.0001057**	ROS/n.e.
8-HDoHE	0.0000566	0.0196	346.29	**1.569e-14**	0.01485	262.37	0.01789	ROS/n.e.
13-HDoHE	0.0000329	0.0112	338.91	**6.276e-15**	0.00896	272.34	n.s.	ROS/n.e.
4-HDOHE	0.00137	0.4325	315.69	**2.879e-10**	0.3605	263.14	n.s.	ROS/n.e.
8-HETE	0.000296	0.0823	278.04	**3.661e-15**	0.0432	145.95	0.005603	ROS/n.e.
16-HDoHE	0.000156	0.0388	248.72	**6.276e-15**	0.03025	193.91	n.s.	ROS/n.e.
8-HETrE	0.000109	0.0245	224.77	**3.984e-10**	0.01745	160.09	n.s.	ROS/n.e.
11-HDoHE	0.000146	0.0217	148.63	**4.434e-10**	0.017	116.44	0.01911	ROS/n.e.
20-HDoHE	0.000123	0.0158	128.46	**3.981e-10**	0.013	105.69	n.s.	ROS/n.e.
10-HDoHE	0.000169	0.01195	70.71	**2.875e-10**	0.00897	53.08	0.04261	ROS/n.e.
9-HODE	0.00593	0.3105	52.36	**9.36 e-10**	0.257	43.34	n.s.	ROS/n.e.
13-HODE	0.0125	0.4955	39.64	**1.582e-09**	0.4375	35.00	n.s.	ROS/n.e.
13-HOTrE(a)	0.000449	0.0123	27.28	**4.785e-13**	0.008075	17.98	n.s.	ROS/n.e.
9-HOTrE	0.000849	0.0163	19.14	**5.496e-10**	0.0110	13.02	n.s.	ROS/n.e.
13-HOTrE(g)	0.00567	0.0432	7.62	**2.519e-09**	0.0308	5.43	n.s.	ROS/n.e.

Supplemental Table S2: median and p-values of LM analysis of NEDA (n=12) and EDA (n=16) patients of the SABA trial at baseline and after SFE treatment. Median data are presented as LM signal intensitiy [peak area]. Following Bonferroni adjustment all p < 0.001136 are significant and highlighted in bold print. For analysis of LM expression at baseline between NEDA and EDA patients, LM with a p ≤ 0.01 are highlighted in grey.

Lipid Mediator	Median of peak area (LM intensity) of NEDA patients at baseline	Median of peak area (LM intensity) of EDA patients at baseline	NEDA versus EDA patients at baseline, p-value	Median of peak area (LM intensity) of NEDA patients after eight months of SFE treatment	SFE treatment NEDA patients: Baseline versus Month 8 p-value	Median of peak area (LM intensity) of EDA patients after eight months of SFE treatment	SFE treatment EDA patients: Baseline versus Month 8 p-value	Key enzyme
5,12-diHETE	2.375	2.74	n.s.	1.71	0.05225	1.845	0.01047	5-LO
Epi/trans LTB4	0.452	0.586	n.s.	0.3395	n.s.	0.38	0.009186	5-LO
5-HEPE	0.3035	0.3275	n.s.	0.219	n.s.	0.1955	0.05245	5-LO
5-HETrE	0.06565	0.1135	0.01302	0.0256	0.01611	0.05125	0.007629	5-LO
5-HETE	2.325	2.955	n.s.	1.350	0.05225	1.655	0.005157	5-LO
LTB4	0.09895	0.1495	n.s.	0.0814	0.021	0.08735	0.006287	5-LO
5-oxo-ETE	0.0533	0.08695	0.04207	0.0339	**0.0004883**	0.0509	0.009186	5-LO
7-HDoHE	0.01175	0.01635	0.01479	0.008675	n.s.	0.01465	n.s.	5-LO
LXA4 isomers	0.1305	0.18	n.s.	0.1028	0.04545	0.111	0.006125	5-LO + 12- or 15-LO
5,15-diHETE	0.08705	0.112	n.s.	0.0571	n.s.	0.0741	0.005157	5-LO+ 12- or 15-LO
LXB4 isomers	0.004145	0.005535	n.s.	0.00257	n.s.	0.00367	0.004181	5-LO+ 12- or 15-LO
RvE1	0.00577	0.007425	n.s.	0.0036	n.s.	0.006965	n.s.	5-LO + COX
PD1	0.007385	0.01295	0.003443	0.007195	n.s.	0.010335	n.s.	12- or 15-LO
PDX	0.00985	0.0156	0.03305	0.008715	n.s.	0.008075	n.s.	12- or 15-LO
15-HETrE	0.0321	0.05605	0.009707	0.0214	n.s.	0.02905	n.s.	12- or 15-LO
15-HEPE	0.006095	0.01175	0.01	0.004045	n.s.	0.00872	n.s.	12- or 15-LO

17-HDoHE	0.01185	0.01715	0.015	0.00818	n.s.	0.0169	n.s.	12- or 15-LO
15-HETE	0.04535	0.09125	0.004297	0.02785	n.s.	0.0482	0.03864	12- or 15-LO
12-HEPE	0.0089	0.01565	0.04588	0.00528	n.s.	0.0127	n.s.	12- or 15-LO
14-HDoHE	0.01275	0.01975	0.02584	0.009105	n.s.	0.0181	n.s.	12- or 15-LO
12-HETE	0.05605	0.119	0.008343	0.04105	n.s.	0.0608	0.02139	12- or 15-LO
PDG2	0.0135	0.0179	0.02256	0.011865	n.s.	0.0125	0.05066	COX
PGE1	0.0128	0.0178	n.s.	0.008045	n.s.	0.01055	0.03864	COX
11-HETE	0.2675	0.504	0.006099	0.1545	n.s.	0.2545	0.05066	COX
PGE2	0.0247	0.03405	0.03662	0.0206	n.s.	0.0215	0.03864	COX
PGD1	0.00398	0.00589	n.s.	0.004245	n.s.	0.00571	n.s.	COX
PGF2a	0.00247	0.00345	n.s.	0.002885	n.s.	0.00278	n.s.	COX
TxB2	0.007	0.00874	n.s.	0.00829	n.s.	0.006745	n.s.	COX
12-HHT	0.0000329	0.00003105	n.s.	0.0000244	n.s.	0.0000279	n.s.	COX
9-HETE	0.07215	0.0923	0.04846	0.04075	0.01611	0.0507	0.004181	ROS/n.e.
8-HDoHE	0.01625	0.02225	n.s.	0.01295	n.s.	0.01885	n.s.	ROS/n.e.
13-HDoHE	0.009615	0.014	0.02256	0.006645	n.s.	0.01295	n.s.	ROS/n.e.
4-HDOHE	0.381	0.4855	n.s.	0.3725	n.s.	0.3515	n.s.	ROS/n.e.
8-HETE	0.0624	0.1325	0.007145	0.0349	n.s.	0.0563	0.0155	ROS/n.e.
16-HDoHE	0.0315	0.0464	0.02256	0.0227	n.s.	0.04205	n.s.	ROS/n.e.
8-HETrE	0.02215	0.0388	0.01386	0.0155	n.s.	0.0208	n.s.	ROS/n.e.
11-HDoHE	0.01845	0.0268	0.02256	0.01305	n.s.	0.0233	0.05066	ROS/n.e.
20-HDoHE	0.0134	0.0188	0.01724	0.009265	n.s.	0.0163	n.s.	ROS/n.e.
10-HDoHE	0.009815	0.01475	0.02288	0.0069	n.s.	0.0145	n.s.	ROS/n.e.
9-HODE	0.252	0.385	0.01479	0.248	n.s.	0.257	n.s.	ROS/n.e.
13-HODE	0.4015	0.5785	0.01479	0.3805	n.s.	0.455	n.s.	ROS/n.e.
13-HOTrE(a)	0.01046	0.014	n.s.	0.006745	n.s.	0.00901	n.s.	ROS/n.e.
9-HOTrE	0.0141	0.02065	0.05114	0.01105	n.s.	0.01325	n.s.	ROS/n.e.
13-HOTrE(g)	0.03445	0.0525	n.s.	0.0304	n.s.	0.0339	n.s.	ROS/n.e.

Supplemental Table S3: Correlations in the total SABA cohort (from Figure 5). Strong correlations are highlighted in blue.

Rank	Parameters (all described as delta change from baseline to Month 8)	Total SABA cohort (n=28)	NEDA RR-MS patients (n=12)	EDA RR-MS patients (n=16)
**NEGATIVE CORRELATIONS**				
# 2	CEL volume during treatment & PGE_2	r = -0.5262 p-value = 0.004023	not significant	not significant
# 3	CEL volume during treatment & LXA_4 isomers	r = -0.5240 p-value = 0.004213	not significant	not significant
# 4	IL-17+ CD4+ T cells & PGE_1	r = -0.4682 p-value = 0.01586	r = -0.7364 p-value = 0.01338	not significant
# 5	PGE_1 & IL-17+ CD4+ T cells	r = -0.4528 p = 0.02018	r = -0.7364 p = 0.01338	-0.3806972 not significant
# 6	CEL volume during treatment & FoxP3	r = -0.4428 p-value = 0.02351	not significant	not significant
# 7	CEL volume during treatment & PGD_2	r = -0.4274 p-value = 0.02329	not significant	not significant
# 9	Serum IL-17 & 12-HETE	r = -0.4243 p-value = 0.07584	not significant	not significant
# 10	PGE_1 & CD40 mDC expression	r = -0.4126 p = 0.03717	r = -0.5545 p-value = 0.08155	not significant
# 11	IL-17+ CD4+ T cells & 4-HDoHE	r = -0.4107 p-value = 0.03713	not significant	not significant
# 12	CEL volume during treatment & $PGF_{2\alpha}$	r = -0.3964 p-value = 0.03679	not significant	not significant
# 13	CD86 expression on myeloid DCs & 16-HDoHE	r = 0.3942 p-value = 0.04724	not significant	r = -0.6045 p-value = 0.01699
# 14	PGE_2 & Serum IL-17	r = -0.3903 p-value = 0.04413	not significant	r = -0.6045 p-value = 0.01699

# 15	IL-17+ CD4+ T cells & 5-oxoETE	r = -0.3895 p-value = 0.04918	not significant	r = -0.5273 p-value = 0.04341
**POSITIVE CORRELATIONS**				
# 1	**NfL & total contrast enhancing lesion volume (CEL)**	**r = 0.7011 p-value = 0.00005129**		
# 2	**IL-17+ CD4+ T cells & CD40 mDC expression**	**r = 0.6105 p-value = 0.0009265**	r=0.5818 p-value = 0.06553	r = 0.6023 p-value = 0.01749
# 3	NfL & IL-10+ CD8+ T cells	r = 0.5630, p = 0.002748		
# 4	CD86 expression on myeloid DCs & 15-HETrE	r = 0.4564 p-value = 0.02009	not significant	r = 0.6643 p-value = 0.008578
# 5	CD86 expression on myeloid DCs & 8-HETrE	r = 0.4414 p-value = 0.02502	not significant	r = 0.7071 p-value = 0.004316
# 6	CD86 expression on myeloid DCs & 12-HETE	r = 0.4332 p-value = 0.0281	not significant	r = 0.7679 p-value = 0.001282
# 7	CD86 expression on myeloid DCs & 5-HETrE	r = 0.4256 p-value = 0.03119	not significant	r = 0.6536 p-value = 0.01003
# 8	IL-17+ CD8+ T cells & 8-HETrE	r = 0.4182 p-value = 0.0335	not significant	r = 0.5214 p-value = 0.04883
# 9	CD86 expression on myeloid DCs & 12-HETE	r = 0.4161 p-value = 0.03552	not significant	r = 0.75 p-value = 0.001907
# 10	CD86 expression on myeloid DCs & 8-HDoHE	r = 0.4133 p-value = 0.03684	not significant	r = 0.7071 p-value = 0.004316
# 11	CD86 expression on myeloid DCs & 15-HEPE	r = 0.4099 p-value = 0.03855	not significant	r = 0.675 p-value = 0.007294
# 12	CD86 expression on myeloid DCs & 11-HDoHE	r = 0.4044 p-value = 0.04141	not significant	r = 0.6893 p-value = 0.005821
# 13	IL-17+ CD8+ T cells & 15-HETrE	r = 0.4038 p-value = 0.04076	not significant	r = 0.5393 p-value = 0.04066
# 14	CD86 expression on myeloid DCs & $PGF_{2\alpha}$	r = 0.4017 p-value = 0.04291	not significant	r = 0.7071 p-value = 0.004316

# 15	CD86 expression on myeloid DCs & 14-HDoHE	r = 0.3969 p-value = 0.04563	not significant	r = 0.7071 p-value = 0.004316
# 16	CD86 expression on myeloid DCs & 13-HDoHE	r = 0.394 p-value = 0.04724	not significant	r = 0.6786 p-value = 0.006901
# 17	IL-17+ CD8+ T cells & 7-HDoHE	r = 0.3929 p-value = 0.04709	not significant	r = 0.5608 p-value = 0.03228
# 18	IL-17+ CD8+ T cells & 10-HDoHE	r = 0.3902 p-value = 0.04879	not significant	r = 0.6179 p-value = 0.01631
# 19	CD86 expression on myeloid DCs & 12-HEPE	r = 0.3901 p-value = 0.04975	not significant	r = 0.6286 p-value = 0.01417

<u>Top 3 correlations for the total SABA patient cohort plotted:</u>

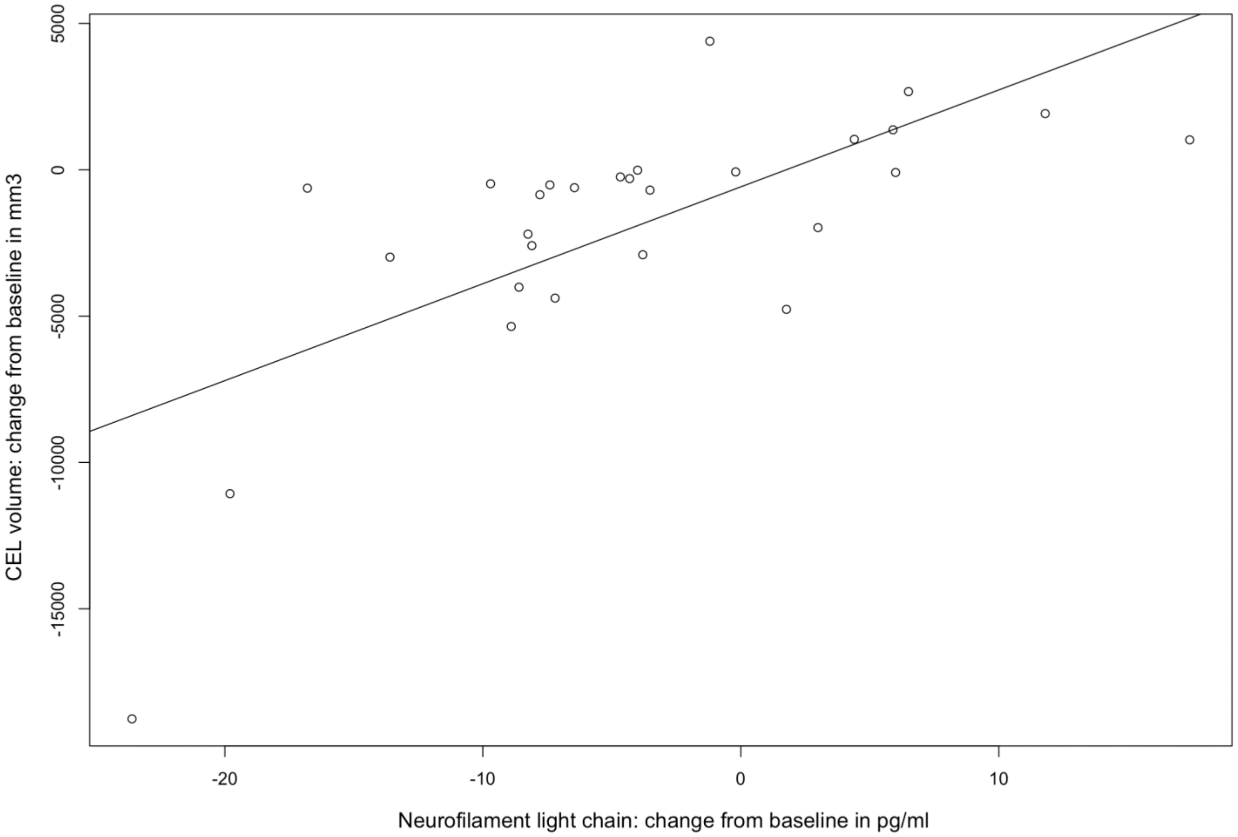

Supplemental Figure 1: NfL concentration & total contrast enhancing lesion volume (CEL): r = 0.7011, p-value = 0.00005129

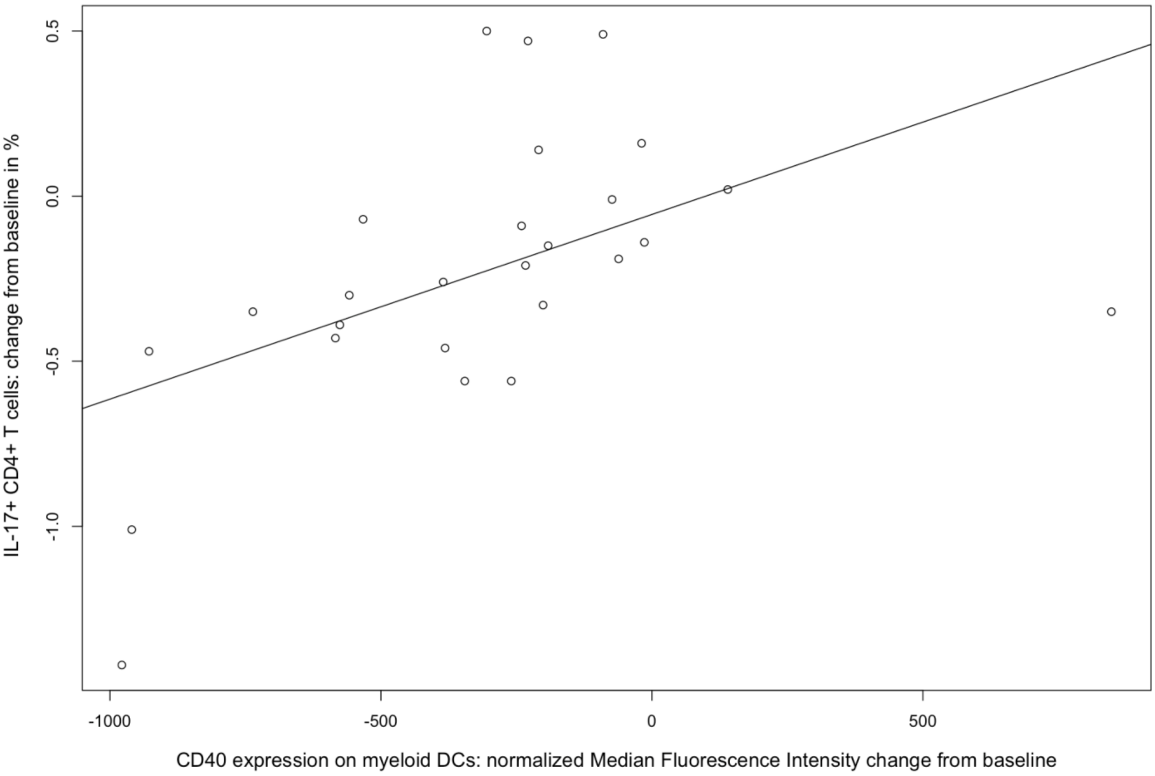

Supplemental Figure 2: IL-17+ CD4+ T cells and CD40 expression on myeloid dendritic cells: r = 0.6105, p-value = 0.0009265

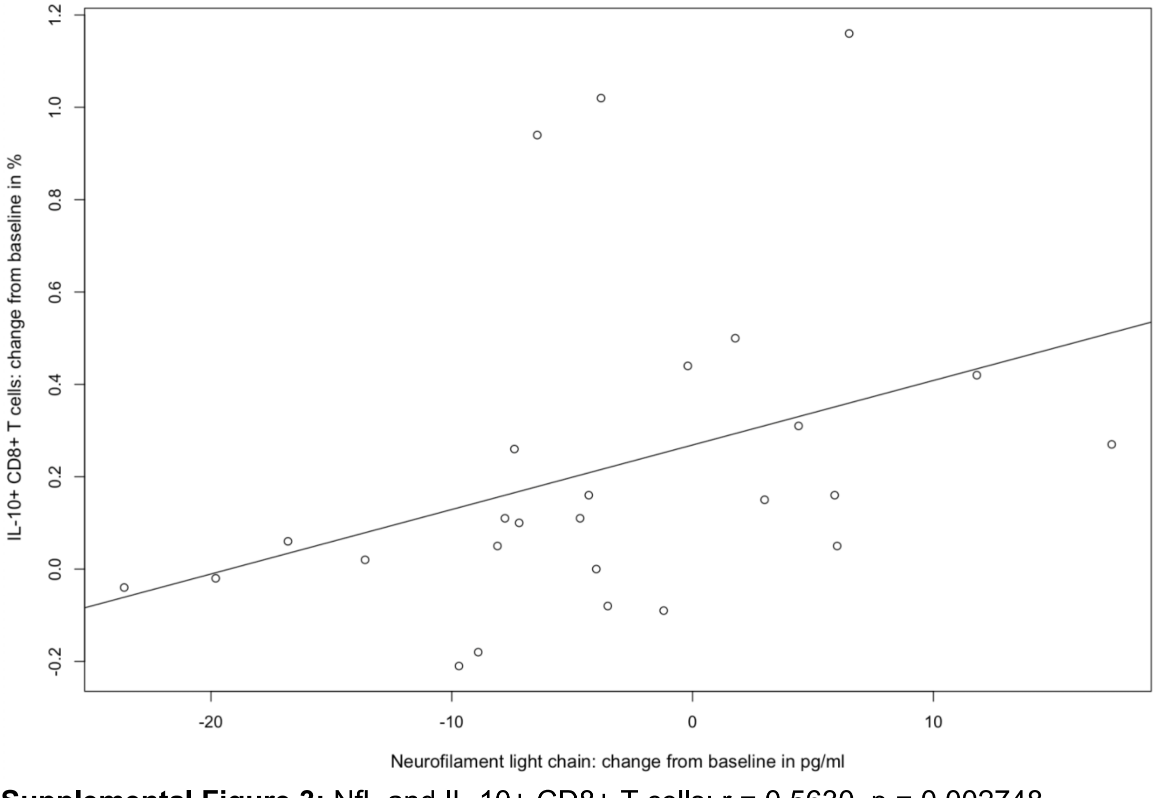

Supplemental Figure 3: NfL and IL-10+ CD8+ T cells: r = 0.5630, p = 0.002748

Supplemental Table S4: Correlations in the NEDA cohort (from Figure 3). Strong correlations are highlighted in green.

	Parameters (all described as delta change from baseline to Month 8)	Total SABA cohort (n=28)	NEDA RR-MS patients (n=12)	EDA RR-MS patients (n=16)
# 1	**CTLA-4 expression & CEL volume**	not significant	**r = -0.8364 p = 0.005952**	not significant
# 2	**PGE1 & IL-10+ CD8+ T cells**	not significant	**r = -0.7455 p-value = 0.01187**	not significant
# 3	**PGE_1 & IL-17+ CD4+ T cells**	-0.4528 p = 0.02018	**r = -0.7364 p = 0.01338**	not significant
# 4	**NfL & 12-HHT**	not significant	**r = -0.7143 p-value = 0.008143**	not significant
# 5	**NfL & 5-HETrE**	not significant	**r = -0.6484 p-value = 0.01958**	not significant
# 6	**NfL & 5-oxo-ETE**	not significant	**r = -0.6374 p-value = 0.02229**	not significant
# 7	**CD86 expression on myeloid DCs & Resolvin E1**	not significant	**r = -0.6455 p-value = 0.037**	**0.55 p-value = 0.03628**
# 8	CEL volume & 12-HHT	not significant	r = -0.5659341 p-value = 0.04734	not significant
**POSITIVE CORRELATIONS**				
# 1	**CTLA-4 expression & 12-HHT**	not significant	**r = 0.7282 p = 0.01105**	not significant
**# 2**	**PGD_2 & FoxP3**	not significant	**r = 0.7273 p-value = 0.01502**	not significant
# 3	**IL-17+ CD4+ T cells & IL-17+ CD8+ T cells**	not significant	**r = 0.7182 p-value = 0.0168**	not significant
	PGE_2 & Foxp3	not significant	r = 0.6090909 p-value = 0.05188	not significant

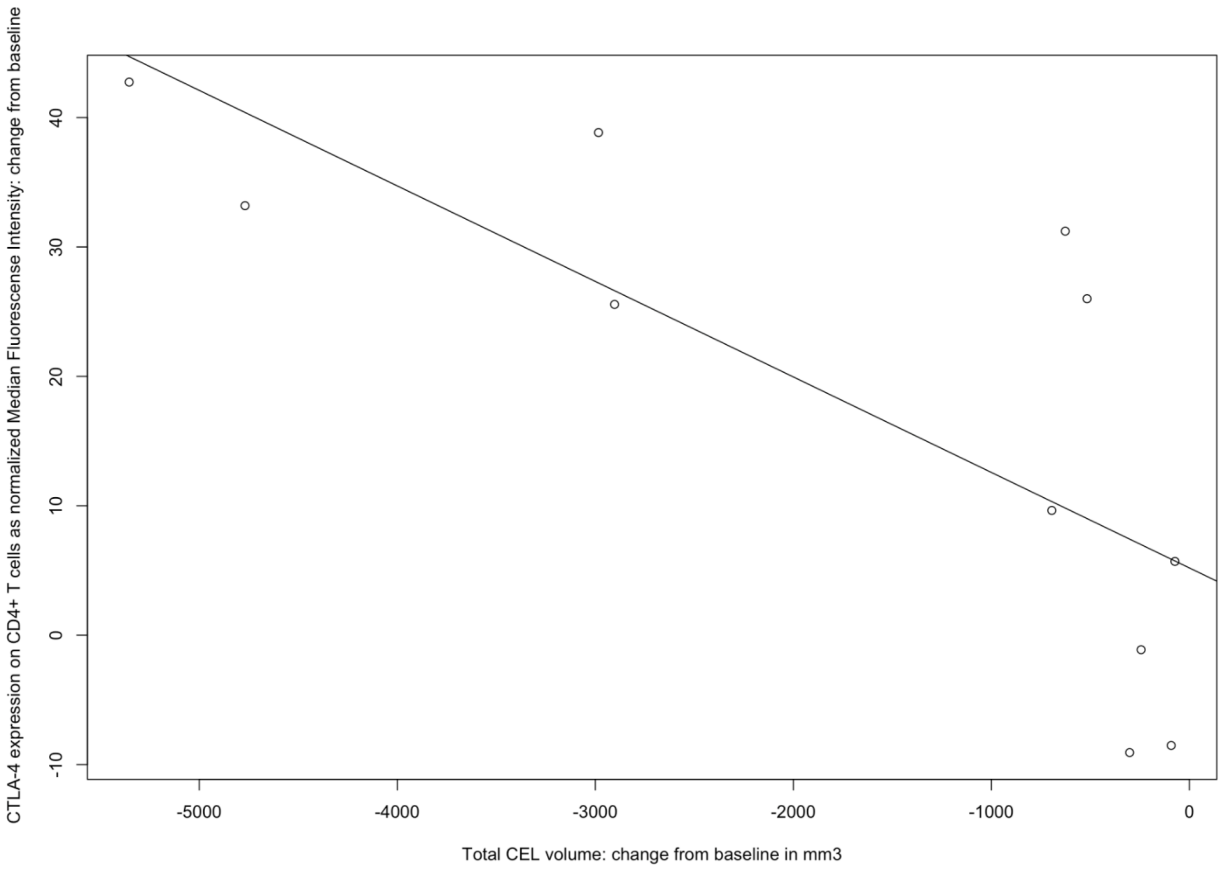

Supplemental Figure 4: CTLA-4 expression & CEL volume: r = -0.8364, p = 0.005952

Supplemental Table S5: Correlations in the EDA group (from Figure 3). Strong correlations are highlighted in orange.

	Parameters (all described as delta change from baseline to Month 8)	Total SABA cohort (n=28)	NEDA RR-MS patients (n=12)	EDA RR-MS patients (n=16)
**NEGATIVE CORRELATIONS**				
# 1	**CTLA-4 expression & IL-17 serum level**	r = - 0.5732 p=0.00274	not significant	**r = - 0.6975, = 0.003842**
# 2	**Serum IL-17 & PGD_2**	not significant	not significant	**r = -0.6153 p-value = 0.01463**
# 3	**PGE_2 & Serum IL-17**	r = -0.3903 -0.3903277	not significant	**r = -0.6045 p-value = 0.01699**
# 4	Serum IL-17 & PGD_1	not significant	not significant	r = -0.5830 p-value = 0.02256
# 5	IL-10+ CD8+ T cells & 12-HHT	not significant	not significant	r = -0.5357 p = 0.03135
# 6	IL-17+ CD8+ T cells & CEL volume	not significant	not significant	r = -0.5357 p = 0.0422
# 7	Foxp3 & 8-HETrE	not significant	not significant	r = - 0.525 p= 0.0471
# 9	IL-17+ CD4+ T cells & 5-oxoETE	-0.3895 p-value = 0.04918	not significant	r = -0.5272 p-value = 0.04341
# 10	Foxp3 & 15-HETrE	not significant	not significant	r = -0.5214, p=0.04883
# 11	IL-17+ CD8+ T cells & IL-10+ CD8+ T cells	not significant	not significant	r = -0.525 p-value = 0.0471
**POSITIVE CORRELATIONS**				
# 1	**TGFβ & 12-HHT**	not significant	not significant	**r = 0.7864168 p-value = 0.0005065**
# 2	**CD86 expression on myeloid DCs & 15-HEPE**	0.4331624 p-value = 0.0281	not significant	**r = 0.7678571 p-value = 0.001282**
# 3	**CD86 expression on myeloid DCs & 12-HEPE**	0.4160684	not significant	**r = 0.75**

		p-value = 0.03552		**p-value = 0.001907**
# 4	**CD86 expression on myeloid DCs & 8-HETrE**	0.4413675 p-value = 0.02502	not significant	**r = 0.7071429 p-value = 0.004316**
# 5	**CD86 expression on myeloid DCs & 17-HDoHE**	0.3969231 p-value = 0.04563	not significant	**r = 0.7071429 p-value = 0.004316**
# 6	**CD86 expression on myeloid DCs & 12-HETE**	0.4017094 p-value = 0.04291	not significant	**r = 0.7071429 p-value = 0.004316**
# 7	**CD86 expression on myeloid DCs & 13-HDoHE**	0.4133333 p-value = 0.03684	not significant	**r = 0.7071429 p-value = 0.004316**
# 9	**CD86 expression on myeloid DCs & 14-HDoHE**	0.4044444 p-value = 0.04141	not significant	**r = 0.6892857 p-value = 0.005821**
# 10	**CD86 expression on myeloid DCs & 16-HDoHE**	0.394188 p-value = 0.04724	not significant	**r = 0.6785714 p-value = 0.006901**
# 11	**CD86 expression on myeloid DCs & 11-HDoHE**	0.4099145 p-value = 0.03855	not significant	**r = 0.675 p-value = 0.007294**
# 12	**CD86 expression on myeloid DCs & 15-HETrE**	0.4564103 p-value = 0.02009	not significant	**r = 0.6642857 p-value = 0.008578**
# 13	**CD86 expression on myeloid DCs & 9-HOTrE**	not significant	not significant	**r = 0.6607143 p-value = 0.009043**
# 14	**IL-17+ CD8+ T cells & 16-HDoHE**	0.4236622 p-value = 0.03102	not significant	**r = 0.6571429 p-value = 0.009527**
# 15	**CD86 expression on myeloid DCs & 5-HETrE**	0.425641 p-value = 0.03119	not significant	**r = 0.6535714 p-value = 0.01003**
# 16	**CD86 expression on myeloid DCs & 8-HDoHE**	0.3900855 p-value = 0.04975	not significant	**r = 0.6285714 p-value = 0.01417**
# 17	**IL-17+ CD8+ T cells & 20-HDoHE**	0.4048555 p-value = 0.04021	not significant	**r = 0.625 p-value = 0.01486**
# 18	**IL-17+ CD8+ T cells & 14-HDoHE**	0.4038297 p-value = 0.04076	not significant	**r = 0.6214286 p-value = 0.01557**
# 19	**IL-17+ CD8+ T cells & 10-HDoHE**	0.3901522 p-value = 0.04879	not significant	**r = 0.6178571 p-value = 0.01631**

# 20	**IL-17+ CD8+ T cells & 13-HDoHE**	0.4103266 p-value = 0.03734	not significant	**r = 0.6071429 p-value = 0.01869**
# 21	**IL-17+ CD4+ T cells & CD40 mDC expression**	r = 0.6104652 p-value = 0.0009265	r = 0.5818182 p-value = 0.06553	**r = 0.6023237 p-value = 0.01749**
# 22	IL-17+ CD8+ T cells & 7-HDoHE	0.3928877 p-value = 0.04709	not significant	r = 0.5607143 p-value = 0.03228
# 23	IL-17+ CD8+ T cells & 15-HETrE	0.4038297 p-value = 0.04076	not significant	r = 0.5392857 p-value = 0.04066
# 24	IL-17+ CD8+ T cells & 8-HETrE	0.4181911 p-value = 0.0335	not significant	r = 0.5214286 p-value = 0.04883
# 25	CD86 expression on myeloid DCs & resolvin E_1	not significant	r = - 0.6454545 p-value = 0.037	r = 0.55 p-value = 0.03628
# 26	TGFβ & 15-HETrE	not significant	not significant	r = 0.5344059 p-value = 0.04014

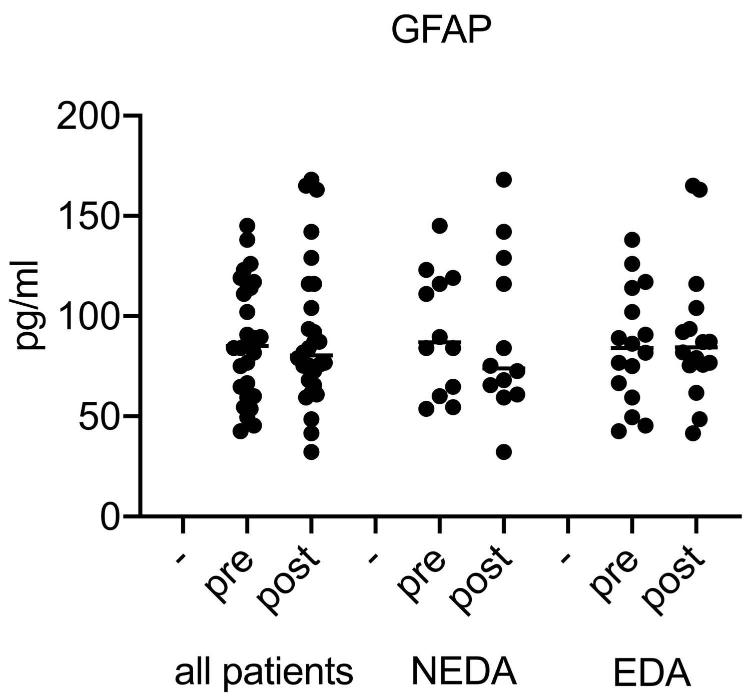

Supplemental Figure 5: GFAP serum levels in RR-MS patients treated with an SFE (n=28) are unaltered.
